# Physiological and Psychological Responses of Viewing a Waterfall Image: A Crossover Study

**DOI:** 10.3390/ijerph20010565

**Published:** 2022-12-29

**Authors:** Hyunju Jo, Harumi Ikei, Yoshifumi Miyazaki

**Affiliations:** Center for Environment, Health, and Field Sciences, Chiba University, 6-2-1 Kashiwa-no-ha, Kashiwa 277-0882, Chiba, Japan

**Keywords:** heart rate variability, natural landscape image, nature therapy, physiological and psychological responses, sympathetic nervous activity, waterfall landscape

## Abstract

Growing interest in the relaxation effect of nature has elicited demands for scientific verification of the various natural elements. This study investigated the physiological and psychological responses of 27 females in their 20 s to viewing a waterfall and urban images (control) presented via a large, high-resolution display for 90 s. High-frequency [HF] for parasympathetic nervous activity and the ratio of low-frequency (LF)/[LF + HF] for sympathetic nervous activity by heart rate variability and heart rate were recorded. Simultaneous changes in oxyhemoglobin concentration in the prefrontal cortex were recorded by near-infrared time-resolved spectroscopy. The modified semantic differential method and Profile of Mood States Second Edition were used to assess the psychological effects on the participants after viewing each image. The results showed that viewing the waterfall image, compared with viewing the urban image, (1) increased sympathetic nervous activity; (2) provided comfortable, relaxed, and natural impressions; (3) improved mood states. In conclusion, visual contact with a waterfall image physiologically activated sympathetic nervous activity and psychologically evoked positive moods and feelings.

## 1. Introduction

It is empirically known that viewing natural landscapes, such as forests, has a stress-reducing and relaxing effect [1]. In recent years, scientific data on the physiological relaxing effects of viewing natural landscapes, determined using physiological indices regarding brain and autonomic nervous activity, has been accumulating. Compared with viewing an urban landscape, sitting in a forest for a short time and viewing a forest landscape have been shown to sedate prefrontal cortex activity [2], enhance parasympathetic nervous activity, and suppress sympathetic nervous activity [3,4,5,6]. However, it is difficult for city dwellers to visit forests, mountains, and other natural wilderness areas frequently because of time and physical constraints. City dwellers are often exposed to natural landscapes through TV and PC displays in daily life.

Previous studies that have assessed natural landscape effects on the physiological state have often used electronic displays and demonstrated that using a visual image with a sense of realism is important for eliciting physiological effects [7]. Recent studies have been conducted using large high-resolution displays that provide a sense of realism to visual stimuli [8,9]. The results showed that prefrontal cortex activity was decreased by viewing a green forest landscape image [8] and viewing a mountain landscape image with autumn foliage increased parasympathetic activity [9], indicating that visual stimulation of forest and mountain landscape images through displays induces physiological relaxation. A previous study investigated viewing videotaped simulated natural landscapes’ physiological effects [10]. The result showed that viewing forest and field landscapes reduced systolic and diastolic blood pressure compared with urban-built landscape scenes. 

Most previous studies have focused on landscapes such as forests, mountains, and fields. Meanwhile, the waterfall landscape is one type of natural landscape. However, the visual stimulation’s physiological effects provided by natural landscapes, such as waterfalls, have not yet been reported.

Therefore, this study aimed to evaluate the visual stimulation effects of a waterfall scene using a large, high-resolution display, with prefrontal cortex activity measured via near-infrared, time-resolved spectroscopy (TRS), and sympathetic and parasympathetic nervous activity assessed via heart rate variability (HRV). Furthermore, the modified semantic differential (SD) and the Profile of Mood States Second Edition (POMS 2) were used to measure and evaluate the psychological effects.

## 2. Materials and Methods

The study’s methods for the physiological and psychological measurements and the experimental design were those described by Jo et al. (2022) [9].

### 2.1. Participants

**Figure 1:**
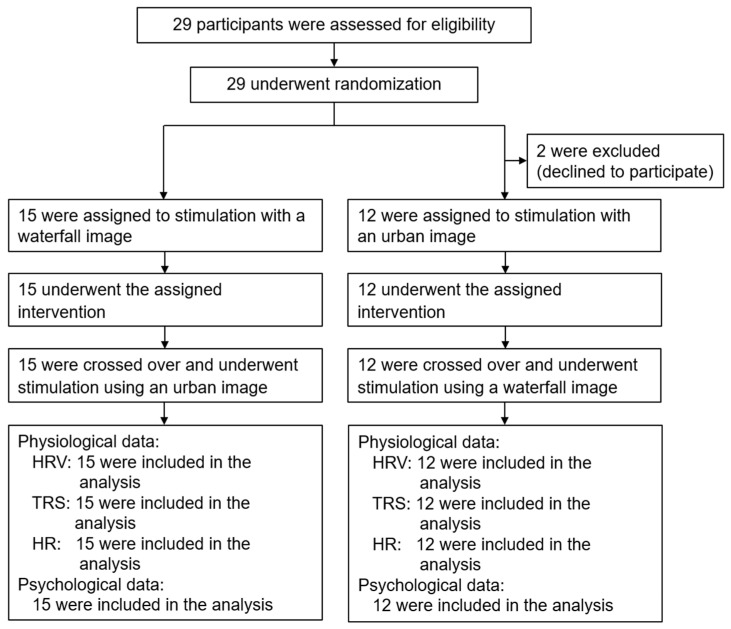
Flowchart of the experimental procedure, including the participant screening, enrollment, follow-up, and analysis flow. HRV (heart rate variability), TRS (near-infrared time-resolved spectroscopy), and HR (heart rate).

In this study, female university students aged 21–29 (mean age ± standard deviation, 23.2 ± 2.4) years participated. Initially, 29 students were scheduled to participate. However, two withdrew on the day of the experiment, resulting in 27 participants. The study used a randomized block design to assign participants to one of two intervention groups with a different viewing order of the images (Figure 1). The mean values of the participants’ physical characteristics were as follows: weight, 48.0 ± 4.4 kg; height, 155.7 ± 4.4 cm; and eyesight, 0.9 ± 0.3 (left), 0.9 ± 0.3 (right), and eyesight included the values corrected by glasses or contact lens (Landolt ring vision, 1.0 equals 20/20) [11]. The exclusion criteria were respiratory disease, habitual smoker, visual acuity of ≤0.3, poor physical condition, and menstruation. The body temperature was measured and confirmed as normal on the day of the experiment. Before the experiment, each participant was placed in a waiting room and given a detailed explanation of the study’s purpose and methods. Participants who agreed to participate signed a consent form. This study was approved by the Ethics Committee of the Center for Environment, Health, and Field Sciences at Chiba University, Japan (approval number: 42). The research protocol was registered in the University Hospital Medical Information Network of Japan (Unique ID: UMIN000039321).

### 2.2. Stimuli

**Figure 2:**
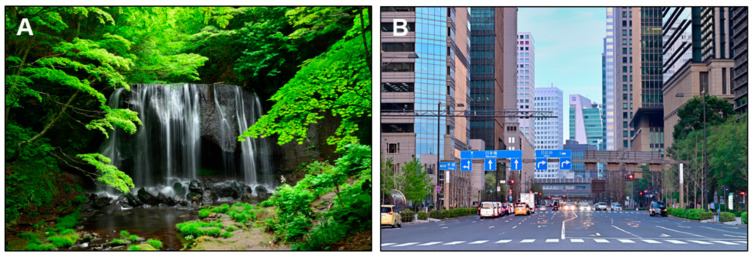
Images used in the visual stimulation. (**A**) Waterfall image: Tatsusawa Fudo Falls, Fukushima. (**B**) Urban image: Eitai Street in Otemachi, Tokyo.

The visual stimuli were photos of a natural waterfall and of a city in Japan. The waterfall image (Figure 2A) shows Tatsusawa Fudo Falls in Fukushima Prefecture. As a control, the urban image (Figure 2B) showed Eita Street in Otemachi, Tokyo. In order to create a sense of realism, the visual stimuli were presented using a large plasma display that was 1872 mm wide × 1053 mm high (85V-type, Panasonic TH-85AX900, Osaka, Japan) with a high resolution of 3840 × 2160 pixels. The distance between the display and the seated participant was 110 cm.

### 2.3. Procedure

Measurements were made in a soundproofed artificial climate chamber wherein temperature (24.0 °C), and relative humidity (50% RH) were held constant. After receiving an overview of the study and objectives in the waiting room, the participants entered the chamber. For physiological measurements, HRV sensors were attached to the participant’s chest, and TRS sensors were attached to their forehead. The experimenter explained the measurement procedure and precautions to each participant seated in a chair. After the room lighting was turned off, a gray image was presented, and physiological responses were recorded during a 60-s resting period. The display was then switched to the waterfall image (Figure 2A) or urban image (Figure 2B), and the physiological responses to the visual exposures were recorded for 90 s. The room lighting was turned on after the physiological measurements. Based on the modified SD method and POMS 2, the psychological questionnaires were then accessed (approximately 120 s). 

After providing the participant a 5 to 10-min break, the same measurement protocol was repeated before and after the viewing of the other image that had not yet been displayed. The study was a within-participants design, and the order of presentation of the waterfall and urban images was counterbalanced to eliminate order effects.

### 2.4. Physiological Indicators

**Figure 3:**
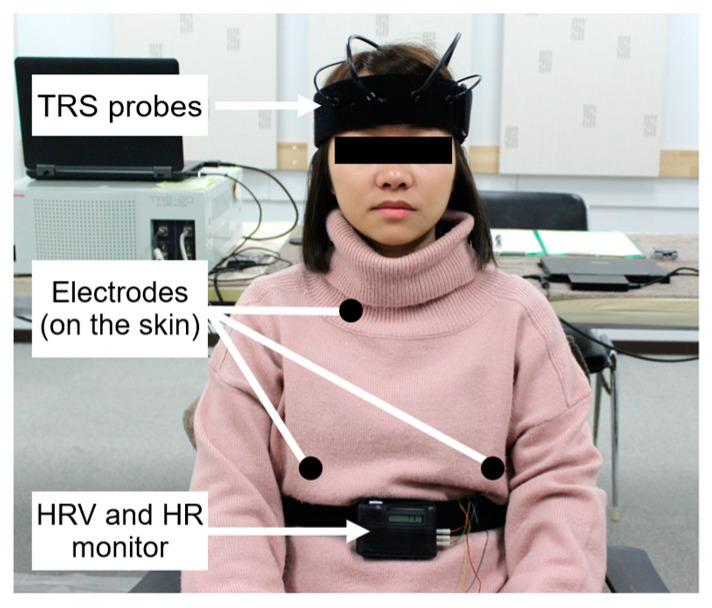
Assessment of physiological indicators. HRV (heart rate variability), HR (heart rate), and TRS (near-infrared time-resolved spectroscopy).

HRV was used as an autonomic nervous activity evaluation. A portable electrocardiograph (Activtracer AC-301A; GMS, Tokyo, Japan) [12,13] was used to measure HRV and heart rate (HR). The maximum entropy method (MemCalc/Win; GMS, Tokyo, Japan) was used to calculate the Power levels of the low-frequency (LF) and high-frequency (HF) [14,15]. HF data were used as an index of parasympathetic nervous activity, and the LF/(LF + HF) ratio was used as an index of sympathetic nervous activity. HF and LF/(LF + HF) data were normalized by conversion to their natural logarithms. TRS was used to evaluate brain activity (TRS-20, Hamamatsu Photonics K.K., Shizuoka, Japan) [16,17] (Figure 3). The physiological values measured during the average 90-s visual exposure were calculated as the difference from the average 30-s rest period before exposure.

### 2.5. Psychological Indicators

First, the impressions evoked by the visual stimulus were examined using the modified SD method [18]. In the study, the three pairs of contrasting adjectives were rated on a 13-point scale. Second, mood states were evaluated according to the answers on the POMS 2 questionnaire with seven subscales regarding mood states [19,20,21]. Further, the total mood disturbance (TMD) score was calculated. We used the POMS 2 short version with 35 questions in the Japanese version to decrease participant burden.

### 2.6. Statistical Analysis

We employed the Statistical Package for the Social Sciences software (version 21.0, IBM SPSS Statistics for Windows, IBM Corp., Armonk, NY, USA) for the statistical analysis. Statistical significance was accepted for *p* < 0.05. For the physiological data of HRV, HR, TRS, and respiratory rate paired *t* tests were used to compare the differences between waterfall (stimulation–rest) and urban (stimulation–rest) images. Cohen’s d (*d*, [22]) was calculated as the effect size for the physiological indices. The Wilcoxon signed-rank test was used to verify the differences in psychological data assessed by the SD method and POMS 2 between the waterfall and urban images. The probability of superiority (*PS_dep_*, [23]) was calculated as the effect sizes of psychological indices.

## 3. Results and Discussion

**Figure 4:**
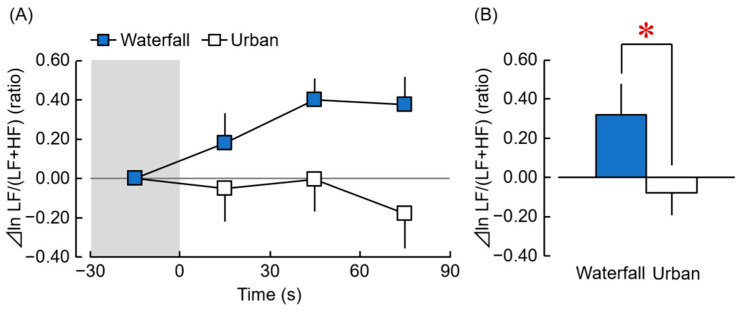
Changes in the ratio of ln LF/(LF + HF) of HRV exposure to the waterfall vs. urban images. (**A**) Changes in the 30 s average ln LF/(LF + HF) over 90 s of exposure (difference from the mean value for 30-s pre-stimulation). (**B**) Changes in the ln LF/(LF + HF) during exposure to the waterfall vs. urban images for 90 s. (*n* = 27 mean ± standard error). * *p* < 0.05, paired *t*-test.

After confirming that there were no significant differences in the respiratory rate between the participants who viewed either a waterfall image or an urban image, a statistical analysis of the HRV data was performed. Figure 4 shows the results of the ratio of ⊿ln LF/(LF + HF), which reflects the sympathetic nervous activity. In the time-dependent changes per 30 s of exposure to the waterfall and urban images over 90 s, the ratio of ⊿ln LF/(LF + HF) while viewing the waterfall image gradually increased. However, while viewing the urban image, the ratio of ⊿ln LF/(LF + HF) almost remained at baseline from 1 to 60 s and then decreased from 61 to 90 s (Figure 4A). A comparison of the overall mean values of the waterfall and urban images for 90-s stimulation (Figure 4B) showed that the ratio of ⊿ln LF/(LF + HF) of the waterfall image was significantly higher than the urban image (Figure 4B, waterfall: 0.32 ± 0.16, urban: −0.08 ± 0.11; t(26) = 2.397, *p* = 0.024, *d* = 0.61). This effect size was considered medium. This finding indicated that viewing the waterfall image increased sympathetic nervous activity compared with viewing the urban image.

Regarding the other indicators of autonomic nervous activity, the ratio of ⊿ln HF, which reflects the parasympathetic nervous activity (waterfall: −0.03 ± 0.12 ms^2^; urban: −0.02 ± 0.10 ms^2^; t(26) = −0.143, *p* = 0.951, *d* = 0.06) and ⊿HR (waterfall: 0.58 ± 0.46 beats/min; urban: −0.44 ± 0.57 beats/min; t(26) = 1.620, *p* = 0.117, *d* = 0.11), no significant differences were noted between viewing the waterfall and urban images.

In the analysis results for the ⊿oxyhemoglobin concentrations on the left (waterfall: −0.15 ± 0.13 μM; urban: −0.39 ± 0.28 μM; t(26) = −0.464, *p* = 0.424, *d* = 0.12) and right (waterfall: −0.24 ± 0.11 μM; urban: −0.15 ± 0.16 μM; t(26) = 0.812, *p* = 0.647, *d* = 0.11) prefrontal cortices, there were no significant differences between the visual stimulation by a waterfall image and by the urban image. 

**Figure 5:**
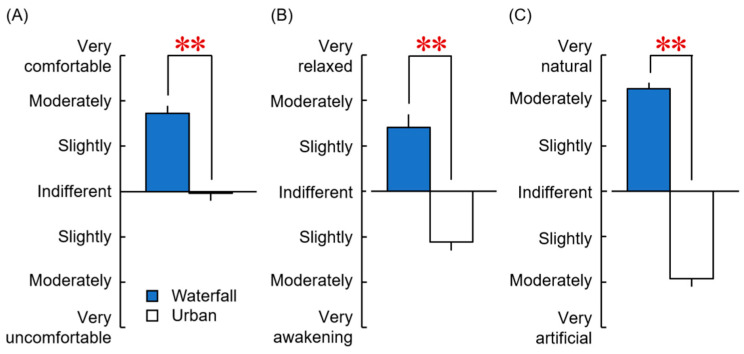
Psychological effects were evaluated using the modified SD method based on three opposing adjective pairs after viewing the waterfall and urban images. (**A**) Comfortable vs. uncomfortable. (**B**) Relaxed vs. awakening. (**C**) Natural vs. artificial (*n* = 27, mean ± standard error). ** *p* < 0.01 (waterfall vs. urban). Wilcoxon signed-rank test.

Figure 5 shows the results of the impression evaluation using three opposing adjective pairs after viewing the waterfall and city images. For the evaluation of comfortable–uncomfortable, the waterfall image was scored slightly to moderately comfortable, whereas the urban image was scored almost equally as comfortable or uncomfortable, indicating that the waterfall image gave a significantly more comfortable impression than the urban image (Figure 5A, *p* < 0.001, *PS_dep_* = 0.926). On the relaxed–awakening evaluation, the waterfall image was scored slightly to moderately relaxed, whereas the urban image was scored as almost slightly awakening, indicating a significant difference between the two images (Figure 5B, *p* < 0.001, *PS_dep_* = 0.852). In the natural–artificial impression evaluation, the waterfall image was rated moderately to very natural, whereas the urban image was rated as an almost moderately artificial impression, with a significant difference between the images (Figure 5C, *p* < 0.001, *PS_dep_* = 1). The findings showed that viewing the waterfall image had psychological effects associated with comfortable, relaxed, and natural impressions compared with viewing the urban image.

**Figure 6:**
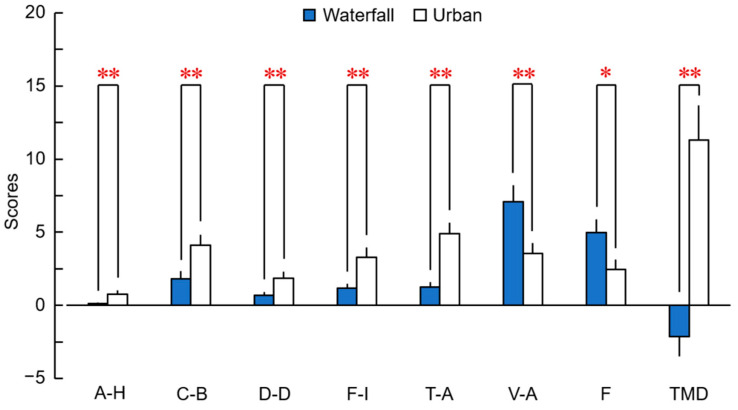
Psychological effects evaluated by POMS 2 after viewing the waterfall and urban images; *n* = 27, mean ± standard error, ** *p* < 0.01, * *p* < 0.05 (waterfall vs. urban), Wilcoxon signed-rank test. A–H, anger–hostility; C–B, confusion–bewilderment; D–D, depression–dejection; F–I, fatigue–inertia; T–A, tension–anxiety; V–A, vigor–activity; F, friendliness; TMD, total mood disturbance.

Figure 6 shows the POMS 2 mood-state results after viewing the waterfall and urban images. On the five negative subscales (mean age ± standard error), anger–hostility [A–H], waterfall: 0.11 ± 0.06, urban: 0.74 ± 0.28, *p* = 0.007, *PS_dep_* = 0.333; confusion–bewilderment [C–B], waterfall: 1.78 ± 0.53, urban: 4.07 ± 0.72, *p* < 0.001, *PS_dep_* = 0.741; depression–dejection [D–D], waterfall: 0.67 ± 0.24, urban: 1.81 ± 0.47, *p* < 0.001, *PS_dep_* = 0.556; fatigue–inertia [F–I], waterfall: 1.15 ± 0.32, urban: 3.25 ± 0.66, *p* < 0.001, *PS_dep_* = 0.667; and tension–anxiety [T–A], waterfall: 1.22 ± 0.35, urban: 4.89 ± 0.75, *p* < 0.001, *PS_dep_* = 0.815), the scores were significantly lower for the visual stimulation by the waterfall image than by the urban image. Conversely, the positive subscale results (vigor–activity [V–A], waterfall: 7.07 ± 1.11, urban: 3.52 ± 0.73, *p* = 0.005, *PS_dep_* = 0.667; friendliness [F], waterfall: 4.96 ± 0.90, urban: 2.44 ± 0.66, *p* = 0.012, *PS_dep_* = 0.593) showed that the waterfall image had significantly higher scores than the urban image. In the evaluation of TMD, the waterfall image had a significantly lower score than the urban image (waterfall: −2.15 ± 1.35, urban: 11.26 ± 2.39, *p* < 0.001, *PS_dep_* = 0.889). These findings showed that viewing a waterfall image via a large display decreased negative-mood states and increased positive-mood states.

In previous nature therapy-related studies, the results were the same as those in the present study with respect to the modified SD method and the shortened version of the POMS 2 [9,24,25], and all findings were suppressed with respect to sympathetic nervous activity [24,26,27,28]. Similar to previous studies, the current study found that comfortable, relaxed, and natural feelings were increased. However, sympathetic nervous activity was increased, which has not been reported previously and could have been caused by the positive change associated with viewing the waterfall. On the shortened version of the POMS 2, the “vigor–activity” score was 7.07 ± 1.11 points, which was a 38% increase relative to the score found in a previous study [9] with an autumn foliage mountain image (5.11 ± 0.77 points) conducted with the same experimental design (Table 1).

Compared with the normal, natural landscape stimuli [8,9], the subjective vigor–activity increased, and sympathetic nervous activity was significantly enhanced by a waterfall image. Selye proposed the concept of “distress” caused by negative and unpleasant emotions and “eustress” caused by positive emotions [29,30], and the waterfall image visual stimuli were interpreted as eustress.

This study had several limitations. Previous studies on nature-derived stimuli have shown a physiologically relaxing effect. In this study, however, the opposite result was obtained as follows: sympathetic nervous activity was increased. Since stimuli derived from the natural environment vary widely, it is necessary to accumulate data using a variety of stimuli in the future to understand the overall picture that natural stimuli’s effects bring on humans. Since the participants were young women in their 20s, further research with subjects having different demographic characteristics, such as males and various ages, should be conducted to enable results generalization. In addition, individual differences in natural stimuli’s physiological effects have been reported [1,5,31], and further verification would be needed.

## 4. Conclusions

This study examined the physiological and psychological effects of viewing waterfall images on a large, high-resolution display. The results showed that stimulation of the waterfall image (1) increased sympathetic nervous activity and (2) produced a positive mood and relaxation compared to the control. In conclusion, visual contact with a waterfall image was shown to produce a physiological awakening and a psychologically relaxing effect.

## Figures and Tables

**Table 1:** POMS 2 scores after stimulation by viewing a waterfall image (*n* = 27) and a mountain image (*n* = 27, Jo et al., 2022 [9]) via a large, high-resolution display.

		A–H	C–B	D–D	F–I	T–A	V–A	F	TMD
Waterfall	Mean ± SE	0.11 ± 0.06	1.78 ± 0.53	0.67 ± 0.24	1.15 ± 0.32	1.22 ± 0.35	7.07 ± 1.11	4.96 ± 0.90	−2.15 ± 1.35
Mountain	Mean ± SE	0.22 ± 0.11	1.67 ± 0.49	0.89 ± 0.28	1.04 ± 0.41	1.52 ± 0.46	5.11 ± 0.77	4.33 ± 0.73	0.22 ± 1.49

Data is shown as the mean ± standard error. A–H, anger–hostility; C–B, confusion–bewilderment; D–D, depression–dejection; F–I, fatigue–inertia; T–A, tension–anxiety; V–A, vigor–activity; F, friendliness; TMD, total mood disturbance.

## Data Availability

The data that support the study findings are available from the corresponding author upon reasonable request.
